# Response of Pasture Grasses to Organic Fertilizer Produced from Black Soldier Fly Frass

**DOI:** 10.3390/plants13070943

**Published:** 2024-03-25

**Authors:** Eoin Rodgers, Elisha Nicolson, Sorcha Lauder, Simon Hodge

**Affiliations:** School of Agriculture & Food Science, University College Dublin, D04 Dublin, Ireland; eoin.rodgers@ucdconnect.ie (E.R.); elishanicolson16@gmail.com (E.N.);

**Keywords:** circular economy, *Hermetia illucens*, improved grassland, Ireland, organic agriculture

## Abstract

Livestock and dairy farmers are increasingly required to maintain productivity and profitability while mitigating the environmental harm associated with high-input agriculture. Accordingly, to reduce reliance on synthetic fertilizers, a wide range of organically derived products are being evaluated for their effects on sward growth and forage quality. This study used glasshouse experiments to investigate the responses of four grass species to a novel organic fertilizer derived from the mass production of black soldier fly larvae [*Hermetia illucens*, HexaFrass™, Meath, Ireland]. Although there was some variability among trials, overall our results indicate that application of HexaFrass produced increased shoot growth of Perennial Ryegrass (PRG; *Lolium perenne* L.), Timothy (*Phleum pratense* L.), and Cocksfoot (*Dactylis glomerata* L.) compared with growth achieved in no-fertilizer control plants. In addition to increases in shoot fresh and dry weight, shoot chlorophyll content was also positively related to the HexaFrass application rate. At nitrogen-equivalent application rates, HexaFrass resulted in lower shoot growth compared with the application of urea, suggesting that the nitrogen contained in HexaFrass may not be immediately plant-available. Of relevance to grazing or silage systems, the addition of HexaFrass resulted in increased shoot regrowth of PRG and Timothy after shoots had been cut. Based on our results, insect-frass-based fertilizers may have a role in low input, organic, and/or regenerative pasture-based livestock systems, although issues may occur due to the relatively high costs and low availability compared with other organic soil amendments such as farmyard manure and slurry.

## 1. Introduction

The agricultural landscape in Ireland is dominated by managed grasslands, designed to provide an abundance of high-quality forage for dairy, beef, and sheep production. These intensive pasture systems are, however, heavily reliant on high inputs of synthetic fertilizers and are therefore associated with significant environmental impacts such as increased greenhouse gas emissions, nutrient leaching, and freshwater eutrophication [1,2,3]. Several aspects of farm management are currently being explored to reduce the environmental footprints of pasture-based livestock systems, including improvement in livestock genetics, modification of sward botanical composition, and the use of legumes within swards to fix atmospheric nitrogen [4,5,6].

To further lower dependence on synthetic fertilizers, various organic amendments, such as manure, slurry, poultry waste, dairy effluent, and composts are being advocated as more holistic approaches to improve pasture soil fertility and maintain pasture productivity [7]. More recently, biofertilizers derived by processing organic materials such as seaweeds, fish waste, and animal byproducts are also being promoted as soil enhancers and plant growth promoters [7]. In addition to supplying macronutrients, these organic amendments can provide additional environmental benefits, such as maintenance of a healthy soil biome and increased soil organic matter, that are commonly associated with organic and regenerative farming systems [8,9].

Relatively recent additions to the organically derived fertilizer market are products based on waste produced by industrial-scale insect farming. The mass rearing of insects, such as meal worms (*Tenebrio molitor* L.) and black soldier flies (BSF; *Hermetia illucens* L.), to produce protein-rich human food or high-quality animal feed provides an excellent example of modern circular economy principles, where biological byproducts, such as brewery or food waste, are diverted from landfill and repurposed as an insect rearing medium [10,11]. In turn, in addition to the primary insect crop, the insect rearing process produces a nutrient-rich byproduct which can then be repurposed as an organic fertilizer [12,13,14]. 

This insect waste material, generally known as ‘frass’, contains a mixture of insect faeces, pupal cases, insect exoskeletons, and remnants of the insect rearing diet, along with a suite of microorganisms [14,15,16]. There is a growing body of evidence that indicates that insect frass fertilizers (IFF) can improve the growth and yield of a wide range of plant groups, from herbs and vegetables to forage plants and cereals [14,17,18,19,20,21]. Recently, several additional benefits of frass-based amendments have been reported, such as improved seed germination, increased shoot nutritional content, enhanced resistance to abiotic and biotic stresses, and improved rhizosphere microbiome [18,20,22,23].

With regard to the effects of IFF on monocotyledonous plants, several publications have described positive responses in growth and/or yield of cereals such as maize [21,24], rice [25], barley [26], wheat, oats, and spelt [20]. Far fewer studies appear to have been performed investigating the effects of IFF on forage grasses. Kebli and Sinaj [27] observed that black soldier fly frass fertilizer (BSFF) had the same growth promoting effect on Perennial Ryegrass (*Lolium perenne* L.) as standard fertilizers, and Menino et al. [28] reported that shoot biomass of Italian Ryegrass (*Lolium multiflorum* Lam.) also increased with application rate of BSFF. Subsequently, Menino et al. [29] suggested that the fertilizing capacity of cattle slurry processed by BSF produced a greater growth response in *L. multiforum* than a fertilizer produced by composting the same slurry in a more traditional manner. Houben et al. [30] reported that mealworm frass increased biomass of *L. multiflorum* and produced similar shoot growth as synthetic NPK fertilizer applied at equivalent rates. Similarly, Kammsteiner et al. [31] reported that BSFF created from three different feedstocks produced similar shoot growth in *L. perenne* to plants receiving the N equivalent as ammonium nitrate. Watson et al. [32], however, suggested that the effects of insect frass on grass performance could be contradictory, inhibiting seed germination, but improving growth and nutrient uptake of shoots. In terms of other forage plants, Hodge and Conway [19] described how BSFF fertilizer produced a positive growth response in chicory and plantain, two herbaceous species often included in multi-species grazing swards. Hodge and Conway [19] and Menino et al. [29] also highlighted that when studying plants designated for livestock forage, it is important to assess the effects of IFF on secondary growth after cutting, as these plants will be grazed or harvested for silage.

Though studies of IFF and forage species are few, they often highlight a number of issues that frequently arise when evaluating the efficacy of IFF. Although plant growth often responds positively to increased IFF application rate, in many cases this trend can be non-linear, with high doses of IFF inhibiting growth or even causing plant mortality [19,20,29]. Other variability in the effects of IFF on plant growth has been observed because of differences in plant potting media [14,20], among plant species [14], and between growing seasons [25]. Inconsistency in the quality and nutrient content of IFF can be caused because of the feedstock, the species of insect being farmed, and the treatments or composting processes employed before the frass is applied to plants or soil [11,24,31,33,34,35,36]. 

Variability among studies has led some authors to suggest that, rather than using a statistical significance approach based on the results of a single trial, evaluations of organically derived fertilizers should be based on a summary of results from several independent investigations [14,37,38,39,40,41]. In these instances, variability in absolute plant growth (due to different growing conditions or plant species) can be mitigated by expressing growth responses as standardized effect sizes, such as Hedge’s *g* and Cohen’s *d* [42]. Additionally, although the effectiveness of IFFs is often determined by comparisons with plant growth obtained under classical, no fertilizer control conditions, it is also necessary to compare changes in plant growth and quality with those obtained with equivalent nutrient rates of standard fertilizers [14,29,31]. In the case of high-input pasture systems, these standard fertilizers would typically be dominated by nitrogen, such as ammonium nitrate or urea. 

The primary objective of this study was to evaluate quantitative and qualitative responses of forage grass species to application of HexaFrass, a commercially available BSFF. Specifically, based on the points raised above, the aims of this study were to: (1) determine the effects of different HexaFrass application rates on shoot growth in two grass species commonly used in Irish pastures (Timothy and Perennial Ryegrass); (2) compare plant responses to HexaFrass with those obtained with a standard nitrogen fertilizer, urea, and another multipurpose organic fertilizer, chicken manure; (3) examine how HexaFrass affects the regrowth of grass shoots after cutting; and, (4) investigate the consistency and repeatability of plant responses across different grass species and within the same grass species across independent trials.

## 2. Materials and Methods

### 2.1. General Methods

Test plants consisted of four grass species commonly used in Irish grazing systems: Perennial Ryegrass (PRG; *Lolium perenne* L.), Timothy (*Phleum pratense* L.), Cocksfoot (*Dactylis glomerata* L.), and Sheep’s Fescue (*Festuca ovina* L.). All seeds were sourced from Fruit Hill Farm, Co. Cork, Ireland. Seeds were sown direct into 9 cm diameter plastic pots and allowed to germinate. After two weeks, seedlings were thinned so that each pot contained three individual plants.

The commercial organically certified fertilizer HexaFrass^TM^ [HF; Hexafly, Co. Meath, Ireland] is produced by rearing black soldier fly (BSF; *Hermetia illucens*) larvae on brewery waste [14]. The frass material is heat treated in order to produce the fertilizer, which typically contains 60% organic matter, is rich in chitin, and has an N–P–K ratio of approximately 4-2-1. Analysis by HexaFly has indicated HF contains other important plant nutrients, such as sulphur (6 g/kg), magnesium (5 g/kg), iron (300 mg/kg), and copper (12 mg/kg) (Alvan Hunt personal communication, 19 October 2021). An aqueous HF solution is approximately neutral (pH 7.3) [20]. In plant growth experiments, the appropriate amount of fertilizer was weighed and added individually to pots prior to the sowing of seeds. 

In order to compare the effects of HF with standard fertilizers, in one experiment (Section 2.2) the effects of HF on PRG and Timothy were compared with those obtained with agricultural-grade urea fertilizer [N-P-K; 46-0-0], which is a commonly used fertilizer on high-input grassland pasture systems. In another experiment (Section 2.4), a commercial chicken manure product (Westland Organic Chicken Manure Pellets; N-P-K 4.5-3.5-2.5) was used as a standard, organically certified, multi-purpose positive control fertilizer. 

The potting mix used in all trials consisted of equal parts by volume of three components: Westland Nutrient Rich Garden Soil, Plagron Coco Bric coir fibre, and vermiculite. This growing medium is considered a low-nutrient potting mix, with prior chemical analysis [Southern Science Laboratories, Kerry, Ireland] indicating N–P–K values of 0.3-0.02-0.5 by dry weight. 

All experiments were conducted in a glasshouse at Rosemount Environmental Research Station, University College Dublin (53.30535, −6.23330), between February and August 2023. During this period the glasshouse had an average daily temperature of approximately 18 °C (range 16–33 °C) and average daily relative humidity of 56% (range 42–83%). No artificial lights were used throughout the trials, and experimental treatments were arranged on glasshouse benches using a randomized design.

Plants were watered with untreated water every 2–4 days, and were always watered the day prior to harvesting. At harvest, the stems and leaves (‘shoot’) were cut at the soil surface, and shoot fresh weight (fwt) obtained (when required). Shoots were placed in paper bags, dried in an oven at 65 °C for 3 days, and then the shoot dry weight (dwt) was obtained. Shoot dry matter content (DM; %) was calculated as 100 × (dwt/fwt).

### 2.2. The Effect of HexaFrass™ Application Rate on PRG and Timothy Shoot Growth and Quality 

To examine how PRG and Timothy responded to increasing amounts of HF, the following rates were applied to each pot: 0, 2, 4, 6, 8, and 12 g. For each of the two grass species, there were ten replicates of the 0 g and 4 g HF treatments, and eight replicates for the other application rates. In terms of N application rates per ha, each 2 g of HF per pot is equivalent to 125 kg N per ha (Supplementary Document S1).

To compare the effects of HF on plant growth with those obtained with an inorganic fertilizer, urea was applied at the following rates per pot: 0.171, 0.343, 0.514, 0.686, and 1.029 g. These rates were selected to represent equivalent nitrogen (N) application rates as the HF treatments listed above, with the assumption that HF contains 4% N and urea consists of 46% N. For both grass species, six replicates of each urea application rate were set up. All plants were harvested 10 weeks after sowing.

### 2.3. The Effect of HexaFrass on Regrowth of PRG and Timothy after Cutting

As PRG and Timothy are used as forage grasses, it was considered important to examine how HF affected the regrowth of both species after shoots had been cut. In this trial, twenty pots containing each grass species were treated with either 0 g or 4 g HF, producing a total of 80 pots. Half of the pots in each treatment were harvested on two occasions: a first cut was performed 7 weeks after sowing and a second cut performed at 10 weeks. The remaining plants were harvested on just one occasion 10 weeks after sowing.

### 2.4. Comparison of HexaFrass with a Standard Multi-Purpose Organic Fertilizer

To further examine the generality of the effects of HF on grass growth, it was desirable to extend the number of grass species tested and to also compare the effects of HF with a standard, multi-purpose organic fertilizer. To meet this objective, HexaFrass (3 g per pot) or chicken manure (3 g per pot) was applied to PRG, Timothy, Cocksfoot, and Sheep’s Fescue, along with a no fertilizer control. There were 10 replicate pots per treatment per grass species. 

### 2.5. An Evaluation of the Variability in the Effect of HexaFrass on Grass Shoot Growth

Previous papers have reported variability in the effects of insect frass and other organic fertilizers on plant performance: it is desirable, therefore, to perform some assessment of the consistency of effects, both within and between studies [14,37]. To meet this objective, four independent trials (labelled Trials#1–#4 for convenience) was each carried out by a different investigator to assess how a single application rate of HexaFrass (3 g per pot) affected shoot dwt of PRG and Timothy compared with a no fertilizer control. Replicate numbers per treatment were: 10 pots for Trial #1, 16 for Trial #2, 10 for Trial #3, and 8 for Trial #4. 

### 2.6. Statistical Analysis

Data were collated and graphics created using Microsoft Excel. All statistical analyses were performed using Genstat v21 (VSN International Ltd., Hemel Hempstead, UK). When different plant species were used in trials, the data for each species were analyzed separately. 

In the examination of HF application rate on shoot weight, polynomial regression curves were initially fitted for each response variable using a quadratic model of the form:

Shoot weight = *a* + *b*(HF) + *c*(HF^2^).

where *a*, *b*, and *c* are constants and HF is the application rate per pot in g. For polynomial relationships, HF_Max_, which is the HF application rate (per pot) that would produce the maximum value of the response variable, can be estimated using the formula −*b*/2*c*. If the polynomial regression indicated that the HF^2^ term was not significant, then this term was removed, and a linear regression was performed.

In the regrowth experiment, the shoot growth in the HF vs. no-HF treatments was initially compared using unpaired *t*-tests, assuming unequal variances. A second analysis comparing the effects of two factors, HF and the cutting regime (1-Cut v 2-Cuts), on total dwt was performed using two-way ANOVA, with separation of the four treatment groups evaluated using least significant differences (*p* < 0.05). In the experiment comparing the effects of HF on shoot growth with those obtained with chicken manure, a one-way ANOVA was used, and pairwise comparisons of treatments made using Fisher’s least significant difference (*p* < 0.05).

For the investigation of consistency among independent trials, shoot dwt with and without 3 g HF was compared within each trial using an unpaired *t*-test assuming unequal variances. To examine for consistency in effect sizes, Hedge’s *g* was calculated as:

$$g=\frac{{\bar{x}}_{1}-{\bar{x}}_{2}}{s}J$$

where *s* is the pooled standard deviation calculated as

$$s=\sqrt{\frac{\left(n_{1}-1\right)s_{1}^{2}+(n_{2}-1)s_{2}^{2}}{n_{1}+n_{2}}}$$

*J* is an adjustment for sample size,

$$J=1-\left(\frac{3}{4\left(n_{1}+n_{2}\right)-9}\right)$$

and $\bar{x}$ = sample mean, *s* = sample standard deviation, and *n* = sample size.

These values of Hedge’s *g* were then tested for heterogeneity by performing a meta-analysis of the trial results using a residual maximum likelihood (REML) procedure, which, in addition to the test statistic for heterogeneity (Q), also produced combined estimates of the overall effect of HexaFrass when assuming the estimates from each trial were fixed or were random.

## 3. Results

### 3.1. The Effect of HexaFrass™ Application Rate on PRG and Timothy Shoot Growth and Quality 

**Figure 1:**
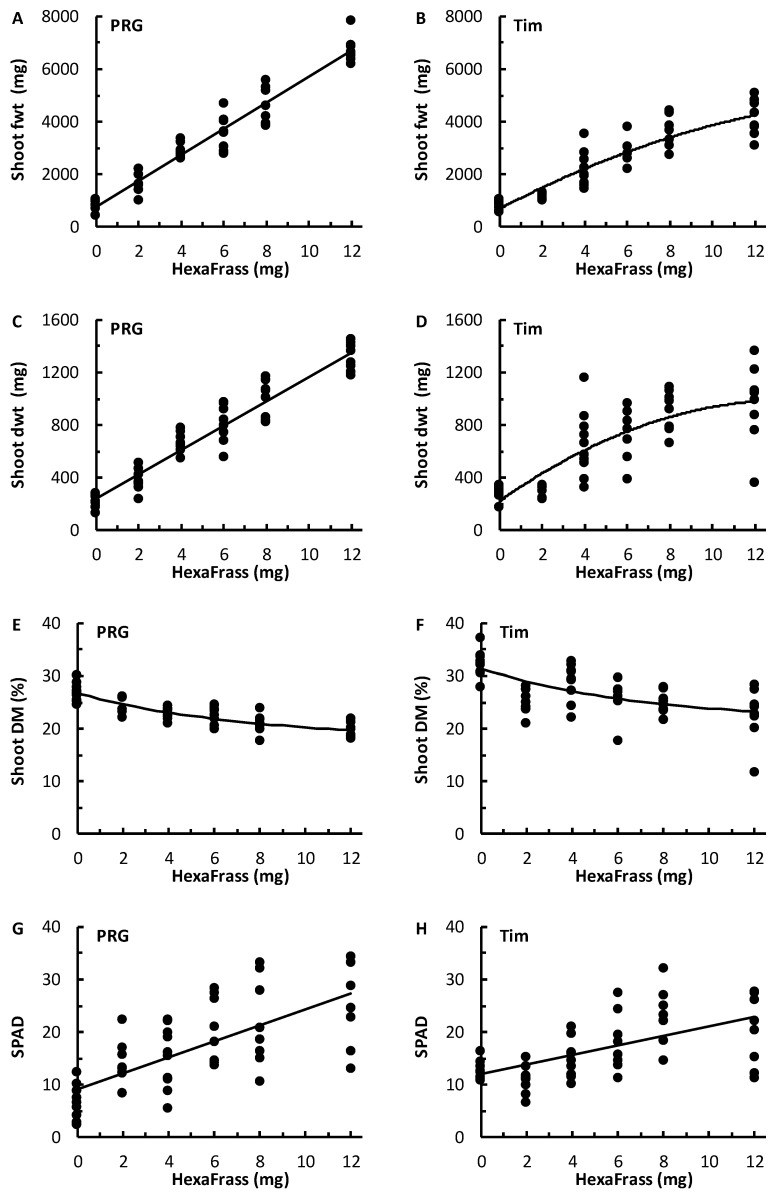
The relationships between HexaFrass application rate (g/pot) and shoot fresh weight (fwt; mg), shoot dry weight (dwt; mg), shoot dry matter content (DM; %), and leaf chlorophyll content (SPAD units) of Perennial Ryegrass (PRG; (**A**,**C**,**E**,**G**)) and Timothy (Tim; (**B**,**D**,**F**,**H**)) grown under glasshouse conditions. For equations of fitted models, see Table 1.

In both PRG and Timothy, shoot fwt and dwt increased with increasing HF application rate (Table 1; Figure 1). For PRG, the relationships between HF application rate and shoot fwt and shoot dwt were linear. For Timothy these patterns could be modelled using quadratic equations, which produced different optimal application HF rates for fwt (20 g HF per pot) and for dwt (13.5 g HF per pot): these rates were both beyond the range of application rates used in this study (Table 1; Figure 1) and were equivalent to field HF application rates of 31 t.ha^−1^ and 21 t.ha^−1^, respectively.

Shoot dry matter content (DM; %) decreased with increasing HF application rate in both grass species (Figure 1). For PRG, DM decreased from a mean of 27% with 0 g HF to a mean of 20% with 12 g HF. For Timothy, DM was slightly higher than for PRG but still decreased from a mean of 32% with 0 g HF to a mean of 23% with 12 g HF. 

In general, shoot chlorophyll content (SPAD units) increased with increasing HF application rate for both grasses. Examination of the scatter plots suggested there could be a levelling off of shoot chlorophyll content at the higher HF doses, and even a decline for Timothy, but the squared terms in the polynomial equations were not statistically significant for either grass species, so the linear models were used (Table 1).

Although we did not aim to compare the performance of the two grass species, it was observed that the variability in the Timothy shoot weights within each HF treatment often tended to be greater than that observed for PRG at the same application rate (Figure 1). Also, the shoot weights of PRG tended to be greater than Timothy, especially at the higher HF application rates (Figure 1). This latter result was partly caused by shoot growth in Timothy beginning to plateau at the higher HF application rates, whereas PRG showed a linear response and continued to gain additional mass with additional HF within these application rates. It was also noted that the 2 g HF treatment for Timothy appeared to produce slightly unusual data, in that the fwt, dwt, and DM were all slightly lower than that predicted by each model (Figure 1).

### 3.2. Comparison of the Effect of HexaFrass on Grass Shoot Growth with That Obtained Using Urea

**Figure 2:**
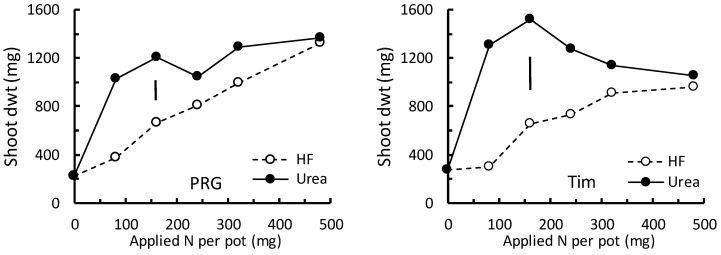
The response of shoot dry weight (dwt) of Perennial Ryegrass (PRG) and Timothy (Tim) to N application (mg per pot) as urea or HexaFrass. To indicate statistically significant differences between pairs of means, the least significant difference (*p* < 0.05) obtained from the nested ANOVA is shown as a vertical bar.

To compare the effects of HF and urea on grass growth, urea was applied at equivalent nitrogen rates (Supplementary Documents S1 and S2). For both PRG (F_5,71_ = 27.2; *p* < 0.001) and Timothy (F_5,71_ = 30.4; *p* < 0.001), there was a statistically significant interaction between the two main factors, application rate and fertilizer type, on shoot growth (Figure 2). Generally, compared with the shoot growth obtained when applying HF, urea resulted in higher shoot dwt at any given N application rate. This effect was most apparent at the lower N application rates and was not apparent at the highest N application rate where shoot growth was very similar for both urea and HF (Figure 2).

The shape of the relationships between shoot dwt and N rate obtained for HF and for urea were not similar. For both PRG and Timothy, the relationships between shoot dwt and urea N application rate were clearly non-linear (Figure 2). For Timothy, mean shoot dwt peaked when around 160 mg urea N was applied to each pot (≅250 kg N.ha^−1^) and then decreased with additional urea N (Figure 2). Conversely, when HF was applied, Timothy shoot dwt did not peak, and this was estimated to occur at approximately 540 mg N by the polynomial model described above (Section 3.1; 13.5 g HF × 4% N). 

For PRG, shoot dwt did not reach a peak with either HF or urea. However, at the lower N application rates, the increase in shoot dwt per additional unit N was much greater with urea than with HF (Figure 2). 

### 3.3. The Effect of HF on Regrowth of Ryegrass and Timothy after Cutting

**Figure 3:**
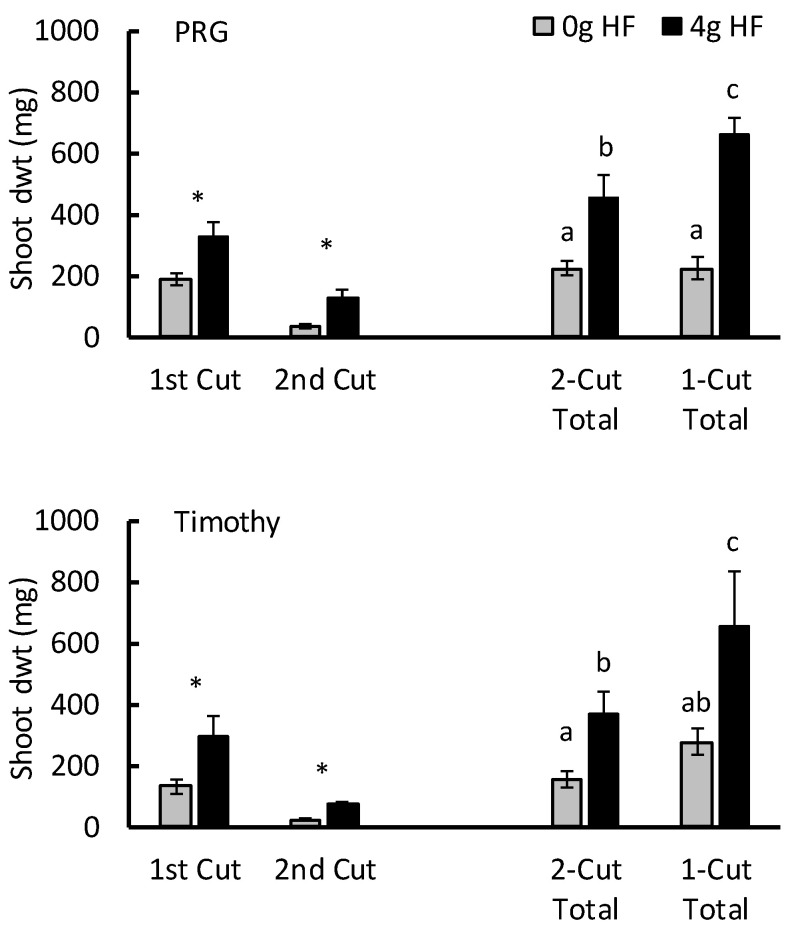
The influence of HexaFrass (0 g and 4 g per pot) on the shoot dry weight growth (mean ± SE) of PRG and Timothy at seven weeks (1st Cut) and after three weeks of regrowth (2nd Cut). The total shoot dwt of the cut plants (2-Cut Total) and plants that were harvested only once after 10 weeks (1-Cut Total) are also presented. Differences between 0 g and 4 g HexaFrass treatments tested by unpaired *t*-tests; * *p* < 0.001. Letter codes for Total cuts indicate separation using Fisher’s least significant difference *p* < 0.05.

The application of 4 g HF significantly increased shoot dwt of PRG and Timothy at both the initial First Cut (7 weeks) and the regrowth Second Cut (10 weeks; Figure 3). When considering the total shoot dwt, there was a significant statistical interaction between the HF treatment and cutting regime for PRG (F_1,36_ = 23.6; *p* < 0.001) but not for Timothy (F_1,36_ = 3.7; *p* = 0.063). However, for both grasses, total shoot dwt was significantly higher when 4 g HF was applied and when the plants were harvested only once (Figure 3).

### 3.4. Comparison of HexaFrass with a Standard Multi-Purpose Organic Fertilizer

**Figure 4:**
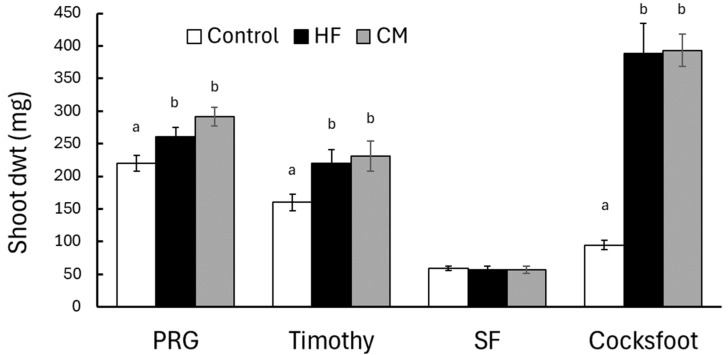
The effect of applying 3 g HexaFrass (HF) or 3 g chicken manure (CM) on the shoot dry weight (mean ± SE) of four grass species: Perennial Ryegrass (PRG), Timothy, Sheep’s Fescue (SF), and Cocksfoot. The control plants received no fertilizer. Within each plant species, treatments with different letter codes indicate significant difference based on pairwise comparisons using Fisher’s least significant difference at *p* < 0.05.

In this experiment, ANOVA identified significant differences among the HF, chicken manure, and no-fertilizer treatments for PRG (F_2,27_ = 10.9; *p* < 0.001), Timothy (F_2,27_ = 3.87; *p* = 0.033), and Cocksfoot (F_2,27_ = 31.3; *p* < 0.001) but not Sheep’s Fescue (F_2,27_ = 0.38; *p* = 0.963) (Figure 4). Compared with the shoot dwt in the no fertilizer control plants, the increase in dwt on addition of 3 g HF was 18% for PRG, 37% for Timothy, and >300% for Cocksfoot (Figure 4). In terms of comparing the effect of HF with the other general organic fertilizer, for all four grass species, the shoot dwt produced by HF was not significantly different to that obtained after application of 3 g of chicken manure.

### 3.5. Variability in the Effects of HexaFrass on Shoot Growth

**Figure 5:**
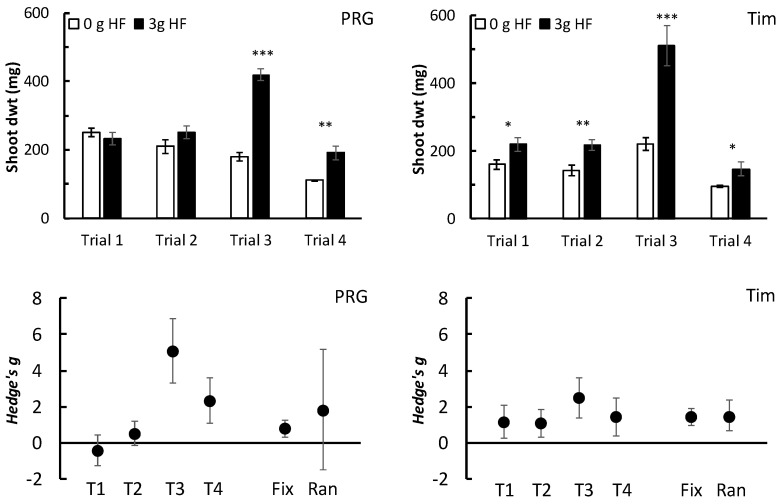
Upper graphs show the effect of applying HexaFrass (3 g per pot) to the shoot dry weight (mean ± SE) of PRG and Timothy in four independent glasshouse trials (Trials #1-#4; T1-T4). *p*-values were obtained using unpaired *t*-test: * *p* < 0.05; ** *p* < 0.01; *** *p* < 0.001. Lower graphs show effect sizes (Hedge’s *g* ± 95% CIs) for each trial (T1–T4) and the combined fixed (Fix) and random (Ran) effect size estimates obtained using a REML meta-analysis procedure.

When considering the variability in effects occurring among different trials with the same grass species, applying 3 g HF significantly increased the shoot dwt of Timothy in all four experiments but in only two of the four experiments for PRG (Figure 5). In Trial #3, the positive effect of adding HF on shoot growth of both PRG and Timothy was considerably more pronounced than in the other trials (Figure 5). Shoot growth was lowest in Trial #4, although the positive effect of HF on PRG and Timothy was still apparent (Figure 5).

In terms of effect sizes, the Hedge’s *g* values obtained for PRG were highly inconsistent and exhibited significant heterogeneity among trials (Q = 36.9 for 3 df, *p* < 0.001) (Figure 5). For Timothy, however, the meta-analysis suggested there was no significant heterogeneity in effect sizes among the four trials (Q = 4.4 for 3 df, *p* = 0.219) (Figure 5). Consequently, both the fixed and random Hedge’s *g* effect sizes for Timothy were significantly different from zero (*p* < 0.001) (Figure 5). For PRG, however, the heterogeneity among the different trials meant that only the fixed effect was significantly different from zero (Hedges *g* = 0.8, *p* < 0.001), but the combined random effect was not (Hedge’s *g* = 1.78, *p* = 0.131) (Figure 5). 

## 4. Discussion

Overall, the results of these glasshouse trials indicated that shoot growth of forage grasses was positively affected by the application of HexaFrass, a black soldier fly fertilizer, compared with plant growth achieved in no fertilizer control plants. Even at the highest application rates used, we observed no negative effects of HF on the germination of seeds [32] or seedling survival [14,19]. Both the initial seedling growth and shoot regrowth after cutting responded positively to HF application, although these positive effects on shoot weight were not observed for all grass species or in all trials. 

Several previous investigations have reported non-linear trends in plant growth in response to increasing application rates of IFF. Unlike in some previous investigations, however, we did not observe significant plant mortality at the highest application rates and thus did not record any ‘zero yields’ [14,19,20]. In the current study, although shoot growth of Timothy was showing indications of levelling off when the maximum application rate of HF (12 g per pot, or the equivalent to 750 kg N.ha^−1^) was applied, the PRG shoot weight was still increasing, suggesting this species may respond positively to even higher application rates. 

The effect of HF on shoot growth of four grass species was comparable with that obtained when applying the same quantity of another multi-purpose organic fertilizer, chicken manure. This finding supports previous results where HF produced similar effects to chicken manure on the growth of herbs and vegetables [14], chicory and plantain [19], and cereals [20]. Adding N-equivalent application rates of urea, however, produced significantly higher shoot growth in PRG and Timothy compared with that obtained with HF. For example, Timothy and PRG had, more or less, reached their maximum shoot growth with urea when 160 mg N was added per pot, but with HF, similar levels of shoot growth in PRG were only achieved when >320 mg N was applied per pot, and were not achieved with Timothy even when the maximum application of 480 mg N per pot was used. These results suggest that a considerable fraction of the N within the HF is not immediately available to the plants. Conversion of organic N in IFF to plant-available forms can be slow and dependent upon microbial abundance and activity, which can affect immobilization and mineralization of frass N over periods lasting several months [30,32,43]. Frass N occurs in several organic forms, such as proteins, exoskeletons, exuviae, and larval excreta, primarily uric acid, and ammonification of these various organic materials leads to release of ammonium ions, which are soluble and available to plants [32]. In terms of soil nutrient content and plant uptake, Menino et al. [28] found that soil P and K were higher in BSFF-treated potting mix, but there was no statistically significant increase in N content. Houben et al. [30] similarly reported that uptake of P and K by *Lolium multiflorum* was similar when plants were treated with IFF compared with synthetic NPK fertilizer, whereas uptake of N was lower with IFF. Future research into the effects of IFF on plant growth and foliage quality would benefit from investigating when and how much of the different nutrients contained within the fertilizer become available to plants, what fraction of these nutrients are actually taken up by plants, and how these processes influence leaching of nutrients out of the system.

The slow release of N from the organic components in IFF may result in positive effects on plant growth occurring over the longer term. In our study, application of HF increased shoot secondary growth after cutting, achieving regrowth over three times that seen in control plants for PRG and over six times the control plants for Timothy. In a long-term pot experiment, Menino et al. [28] also reported increased shoot weight of ryegrass (*Lolium multiflorum*) treated with BSFF after multiple cuts five weeks apart. Hodge and Conway [19] reported similar findings for forage herbs, with HF application resulting in shoot regrowth three times that of the control treatments in chicory and twice that observed in the control plantain seedlings. 

With respect to qualitative changes in grass foliage, we found that application of HF increased the chlorophyll content of PRG and Timothy, an effect that has also been observed for other plants such as cereals, maize, and pak choi [20,24,44]. Chlorophyll production can be limited by availability of nitrogen and other nutrients, and so it seems likely that the positive relationship between HF and SPAD readings arose because of direct nutrient provision. This hypothesis is supported by the chlorophyll content of Timothy and PRG leaves also being positively related to urea application rate (Supplementary Document S2). Increases in leaf chlorophyll can be associated with an increase in photosynthetic capacity and plant growth potential in addition to being a valuable indicator of plant nutritional status and general plant health. Previous studies have indicated that application of BSFF can increase nutrient content (e.g., N–P–K) of grasses and cereal shoots [20,29], and, together with the effects on shoot dry weight and regrowth, these findings suggest that insect-derived fertilizers could increase both the quantity and quality of forage available to livestock.

Although we have performed multiple trials and identified some consistent results, we concede that our experimental system has several limitations: for example, plants were grown in small containers, under benevolent glasshouse conditions, and were grown for only a relatively short time period. While small-scale pot trials such as these clearly have value as a means of high-throughput screening of new fertilizer products on a range of test plants and under different conditions, only tentative suggestions can be made regarding how results might extrapolate to field conditions. For example, based on previous work [14,19,20], a typical HF application rate employed in these glasshouse trials is around 4 g per pot, equivalent to ~6 t HF.ha^−1^. Although this application rate may initially seem high, it is similar to the application rates used in other pot experiments investigating IFF (e.g., 5–10 t. ha^−1^, [30]) and typical application rates for other manures (chicken, cattle, farmyard) on farmland (e.g., 2–20 t. ha^−1^, [45]). Major obstacles for the use of IFF as the primary mode of pasture nutrient management may not be the estimated required application rate but the relative costs compared with other manures, variability among brands and batches of the same material, and the limited supply available due to the very recent intensification and expansion of insect farming.

An important finding from these glasshouse trials is that, even given the relatively stable environmental conditions and absence of biotic and abiotic stresses, we still identified considerable variation among trials in terms of the effects of IFF on plant growth. Previously, systematic variability in the response of plants to IFF has been attributed to plant species, basal nutrient levels, soil type, farmed insect species, and the feed stock used in the insect diet [11,14,19,20,24,31]. In the current study, we also identified variability in trial results with the same plant species grown under, putatively, very similar growing conditions. Very few studies report on the repeatability of results with IFF, but when repeated trials are performed, often some variability is observed regarding the occurrence, or magnitude, of any effects (e.g., [14,25]). Previously, several authors have advocated for repeated testing of novel plant growth promoting products and organically derived fertilizers in order to gauge the robustness of effects and calculate the typical magnitude of effect sizes [14,37,38,39,40,41]. The statistical analysis performed in the current study suggests that the calculation of such standardized effect sizes, such as Cohen’s *d* and Hedge’s *g*, can be a highly useful tool in this regard. Used along with a simplistic meta-analysis procedure, such as REML, this process provides an additional tool to estimate average effect sizes and quantify heterogeneity among trial results. 

## 5. Conclusions

Overall, the results of our glasshouse trials indicate that forage grass species show improved shoot growth after application of HexaFrass, a commercial IFF produced from black soldier fly waste in Ireland. In general, shoot growth was positively related to IFF application rate and was similar to that produced by another chicken-manure-based organic fertilizer but was not as great as that produced by urea applied at equivalent N doses. Secondary growth of grass shoots after cutting was also increased by the IFF which is important for pasture plants that will be grazed or cut for silage. There was some inconsistency among trials in terms of plant responses to IFF. We are uncertain as to why this variability occurred, but it may have been caused by inconsistency among batches of fertilizer and/or experimenter variability. Nevertheless, these results highlight that when evaluating IFF and other organically-derived fertilizers, it is important to ascertain repeatability and consistency of effects across plant species and under variable conditions. Overall, the results indicate HF may have good potential as an organically acceptable soil amendment for low-input pasture systems. Additional research is required to ascertain plant growth responses to HF under field conditions, the economic and long-term benefits of HF application, and the effects of HF on a wider range of forage plant species in monoculture and in mixtures.

## Figures and Tables

**Table 1:** Equations describing relationships between PRG and Timothy response variables with HexaFrass (HF) application rate (g per pot). See Figure 1 for scatter plots.

Grass Species	Response	Relationship	*r* ^2^
PRG	Shoot fwt (mg)	761.2 + 496.9HF	0.95
	Shoot dwt (mg)	239.6 + 92.7HF	0.93
	Shoot DM (%)	18.0 + (8.7 × 0.873^HF^)	0.70
	SPAD	9.2 + 1.5HF	0.50
Timothy	Shoot fwt (mg)	683.6 + 242.2HF − 10.6HF^2^	0.85
	Shoot dwt (mg)	222.6 + 113.4HF − 4.2HF^2^	0.65
	Shoot DM (%)	21.3 + (10.1 × 0.870^HF^)	0.34
	SPAD	12.0 + 0.9HF	0.36

## Data Availability

Data are available on request from the corresponding author.

## Supplementary Materials

The following supporting information can be downloaded at: https://www.mdpi.com/article/10.3390/plants13070943/s1, Supplementary Document S1: Conversion table for HexaFrass (HF) treatments (grams per 9 cm diameter pot) to kg per hectare, and kg N per hectare. N equivalent urea treatments are also given. Area of open end of a 9 cm diameter pot was rounded to 64 cm^2^. Supplementary Document S2: The relationships between Urea application rate (g/pot) and shoot fresh weight (fwt; mg), shoot dry weight (dwt; mg), shoot dry matter content (DM; %) and leaf chlorophyll content (SPAD units) of Perennial Ryegrass and Timothy (Tim; B, D, F, H) grown under glasshouse conditions. For equations of fitted models see Table below.
