# Supplementary material for: Response of Pasture Grasses to Organic Fertilizer Produced from Black Soldier Fly Frass
Source: Plants (Basel). 2024 Mar 25;13(7):943. doi: 10.3390/plants13070943 (PMC11013756; doi:10.3390/plants13070943)
Supplement: Supplementary file 1 [file plants-13-00943-s001.zip › plants-2939160-supplementary.pdf]

plants

MDPI

Supplementary Document S1. Conversion table for HexaFrass (HF) treatments (grams per 9 cm diameter pot) to kg per hectare, and kg N per hectare. N equivalent urea treatments are also given. Area of open end of a 9 cm diameter pot was rounded to 64 cm^2.

HF (g/pot)	HF (kg/ha)	Urea (g/pot)	Urea (kg /ha)	N (g/pot)	N (kg/ha)
0	0	0	0	0	0
2	3125	0.17	272	0.08	125
4	6250	0.35	543	0.16	250
6	9375	0.52	815	0.24	375
8	12500	0.70	1087	0.32	500
12	18750	1.04	1630	0.48	750

Supplementary Document S2. The relationships between Urea application rate (g/pot) and shoot fresh weight (fwt; mg), shoot dry weight (dwt; mg), shoot dry matter content (DM; %) and leaf chlorophyll content (SPAD units) of Perennial Ryegrass and Timothy (Tim; **B**, **D, F, H**) grown under glasshouse conditions. For equations of fitted models see Table below.

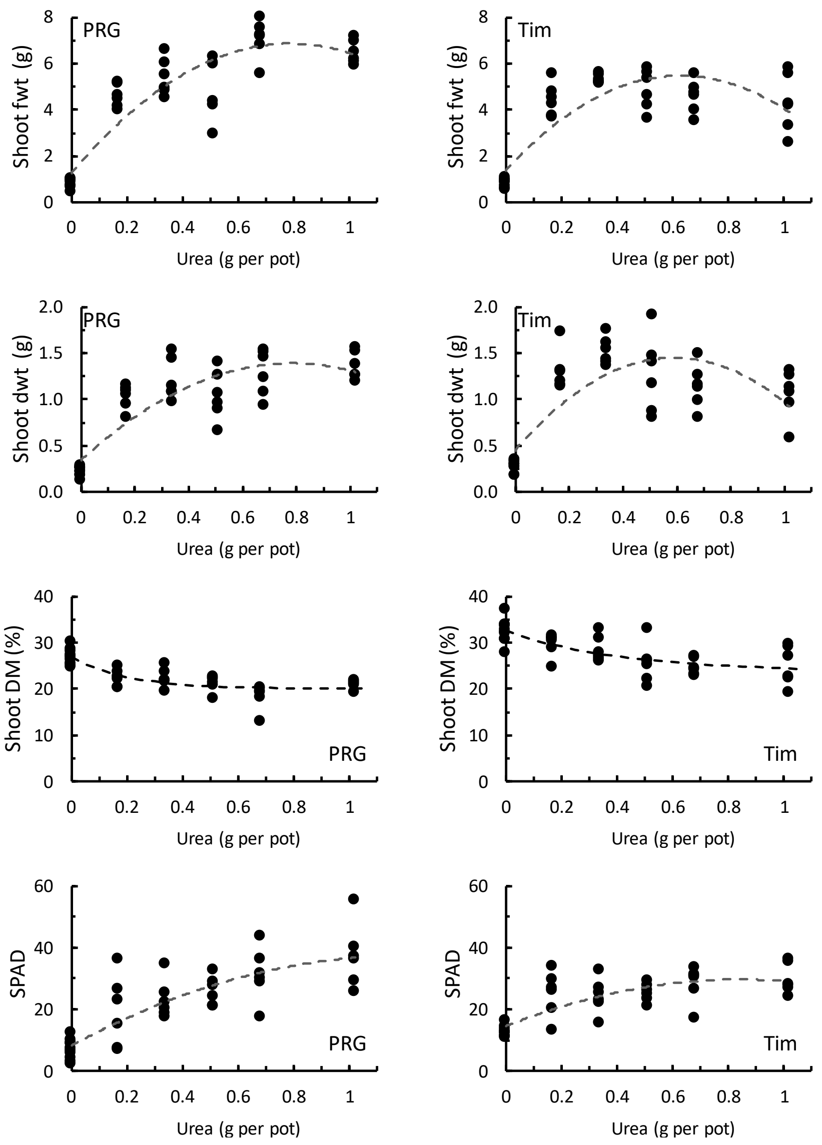

Grass	Response	Fitted model	r^2
PRG	Shoot fwt (mg)	1255.7 + 14288UR – 9089.1UR2	0.85
	Shoot dwt (mg)	342.7 + 2671UR – 1701.9UR2	0.74
	Shoot DM (%)	$20.1 + (6.83 \times 0.006^{UR})$	0.63
	SPAD	$8.15 + 49UR – 20.6UR^2$	0.67
Timothy	Shoot fwt (mg)	1380 + 13052UR – 10380UR2	0.72
	Shoot dwt (mg)	449.7 + 3391.4UR – 2870.8UR2	0.58
	Shoot DM (%)	$23.7 + (8.84 \times 0.086^{UR})$	0.49
	SPAD	$14.7 + 36.1UR – 21.4UR^2$	0.57
